# Analysing the oviposition behaviour of malaria mosquitoes: design considerations for improving two-choice egg count experiments

**DOI:** 10.1186/s12936-015-0768-2

**Published:** 2015-06-20

**Authors:** Michael N Okal, Jenny M Lindh, Steve J Torr, Elizabeth Masinde, Benedict Orindi, Steve W Lindsay, Ulrike Fillinger

**Affiliations:** Disease Control Department, London School of Hygiene and Tropical Medicine, Keppel Street, London, WC1E 7HT UK; International Centre of Insect Physiology and Ecology, Thomas Odhiambo Campus, Mbita, 40305 Kenya; Royal Institute of Technology, 100 44 Stockholm, Sweden; Warwick Medical School, University of Warwick, Coventry, CV4 7AL UK; Liverpool School of Tropical Medicine, Liverpool, UK; International Centre of Insect Physiology and Ecology, Biostatistics Unit, Nairobi, 00100 Kenya; School of Biological and Biomedical Sciences, Durham University, Durham, DH1 3LE UK

**Keywords:** *Anopheles gambiae*, Oviposition, Breeding site, Sample size, Two-choice, Skip oviposition, Cage bioassay

## Abstract

**Background:**

Choice egg-count bioassays are a popular tool for analysing oviposition substrate preferences of gravid mosquitoes. This study aimed at improving the design of two-choice experiments for measuring oviposition substrates preferences of the malaria vector *Anopheles gambiae* senso lato, a mosquito that lays single eggs.

**Methods:**

In order to achieve high egg-laying success of female *An. gambiae* sensu stricto (s.s.) and *Anopheles arabiensis* mosquitoes in experiments, four factors were evaluated: (1) the time provided for mating; (2) the impact of cage size, mosquito age and female body size on insemination; (3) the peak oviposition time; and, (4) the host sources of blood meal. Choice bioassays, with one mosquito released in each cage containing two oviposition cups both with the same oviposition substrate (100 ml water), were used to measure and adjust for egg-laying characteristics of the species. Based on these characteristics an improved design for the egg-count bioassay is proposed.

**Results:**

High oviposition rates [84%, 95% confidence interval (CI) 77–89%] were achieved when 300 male and 300 blood-fed female *An. gambiae* s.s. were held together in a cage for 4 days. The chances for oviposition dropped (odds ratio 0.30; 95% CI 0.14–0.66) when human host source of blood meal was substituted with a rabbit but egg numbers per female were not affected. The number of eggs laid by individual mosquitoes was overdispersed (median = 52, eggs, interquartile range 1–214) and the numbers of eggs laid differed widely between replicates, leading to a highly heterogeneous variance between groups and/or rounds of experiments. Moreover, one-third of mosquitoes laid eggs unequally in both cups with similar substrates giving the illusion of choice. Sample size estimations illustrate that it takes 165 individual mosquitoes to power bioassays sufficiently (power = 0.8, p = 0.05) to detect a 15% shift in comparative preferences of two treatments.

**Conclusion:**

Two-choice egg count bioassays with *Anopheles* are best done with a two-tier design that (1) implements a parallel series of experiments with mosquitoes given a choice of two identical substrates choices and, (2) uses a single mosquito in each test cage rather than groups of mosquitoes to assess the preference of a test or control solution. This approach, with sufficient replication, lowers the risk detecting pseudopreferences.

## Background

*Anopheles* mosquitoes are efficient and resilient vectors of human malaria and filariasis in Africa. These vectors are mainly controlled by extensive use of long-lasting insecticidal nets (LLINs) and indoor residual spraying (IRS) of houses [1]. The two interventions exploit the tendency of *Anopheles funestus* senso lato (s.l.) and *Anopheles gambiae* s.l., the major vectors of malaria in sub-Saharan Africa, to bite and rest indoors [2] and have together contributed to a remarkable and consistent decline in the transmission of malaria through the last decade [3–6]. However, like all vector control interventions, these too have limitations and when used in isolation could fall short in areas with: (1) strains selected for physiological resistance to insecticides [7–10]; (2) secondary vector species that live and bite outdoors [11, 12]; and, (3) cryptic vector sub-groups that bite in the early evening and/or bite outdoors [13]. These and a complex of other factors, including increasing drug resistance and high costs of interventions, make malaria resurgence a grim reality [14]. New strategies with novel tools that combine with LLINs and IRS to target these elusive groups of vectors in addition to the major vectors could prevent the resurgence of disease and hasten malaria elimination.

Larval source management (LSM) can be a complementary intervention for targeting all strains of malaria vectors irrespective of their state of insecticide resistance or resting and biting tendency. However in areas with extensive oviposition sites LSM becomes challenging [15, 16]. Attempts to target oviposition sites by identifying precisely the physical features of water bodies with mosquito larvae have so far been unsuccessful [17, 18]. Nevertheless field studies suggest that the presence of early instar larvae in water bodies is non-random, which may indicate that gravid females select particular water bodies in which to lay their eggs. These studies imply that favourable aquatic oviposition sites though highly heterogeneous in form, space and time [17–20] will display key features that act as signature cues for gravid mosquitoes seeking to lay eggs. Identifying the cues that elicit oviposition behaviour could aid the targeting of larvicides into productive mosquito oviposition sites and allow the development of odour-baited gravid mosquito traps for *Anopheles*.

Laboratory experiments within insect cages are a simple first step in identifying cues that guide short-range habitat selection in gravid mosquitoes [21]. Of these experiments, choice egg-count bioassays are the most common and have been used to search for cues that are preferred or avoided by mosquitoes seeking to lay eggs [22, 23]. Here eggs or egg rafts laid in test substrates by groups of mosquitoes are counted and compared to those laid in a reference substrate, the control. Using these choice tests, chemicals that influence oviposition have been identified for *Stegomyia* [24–26], *Culex* [25, 27–29], and recently for *An.gambiae* sensu stricto (s.s.) [30, 31]. In addition egg-count bioassays have been used to investigate the response of gravid *An. gambiae* s.s. to bacterial cultures [32–34] with variable outcomes. All choice egg-count experiments with *An. gambiae* s.l. have been prepared, implemented and reported in a unique way making it difficult to generalize findings.

Already McCrae raised concern [35] that the very low mean numbers of eggs commonly reported in many choice egg-count experiments with groups of *An. gambiae* s.l. mosquitoes suggest that very few test mosquitoes actually lay eggs and that “the behaviour of only three or four mosquitoes was [therefore] tested”. This could lead to invalid conclusions, should it be true. In addition, it must be considered that putative oviposition substrates may consist of age-dependent organic infusions or concentration-sensitive chemicals, which degrade in time and should be evaluated over a short period. This study sought to improve on the preparation and establish the peak oviposition time of gravid mosquitoes to ensure that most laid eggs during experiments and were tested when they are likely to be most receptive to cues important for oviposition.

It was hypothesized that the egg-laying behaviour of *An. gambiae* s.l. makes it necessary to re-design choice egg-count bioassays uniquely for this species [36]. The need to consider the design of these bioassays for species that lay single eggs and exhibit skip oviposition has been well shown by Chadee and Corbet [37] who proposed a new study design for *Aedes*: one that entailed “recording the distribution of eggs by individual females initially provided with an array of identical sites”. However, their work has been ignored in consequent studies with this genus [38–42]. The present study therefore aimed to present new approaches for: (1) optimizing egg laying in test insects; (2) implementing tests to compare two substrates; and, (3) analysing and reporting finding of egg-count experiments.

## Methods

### Study site

**Figure 1:**
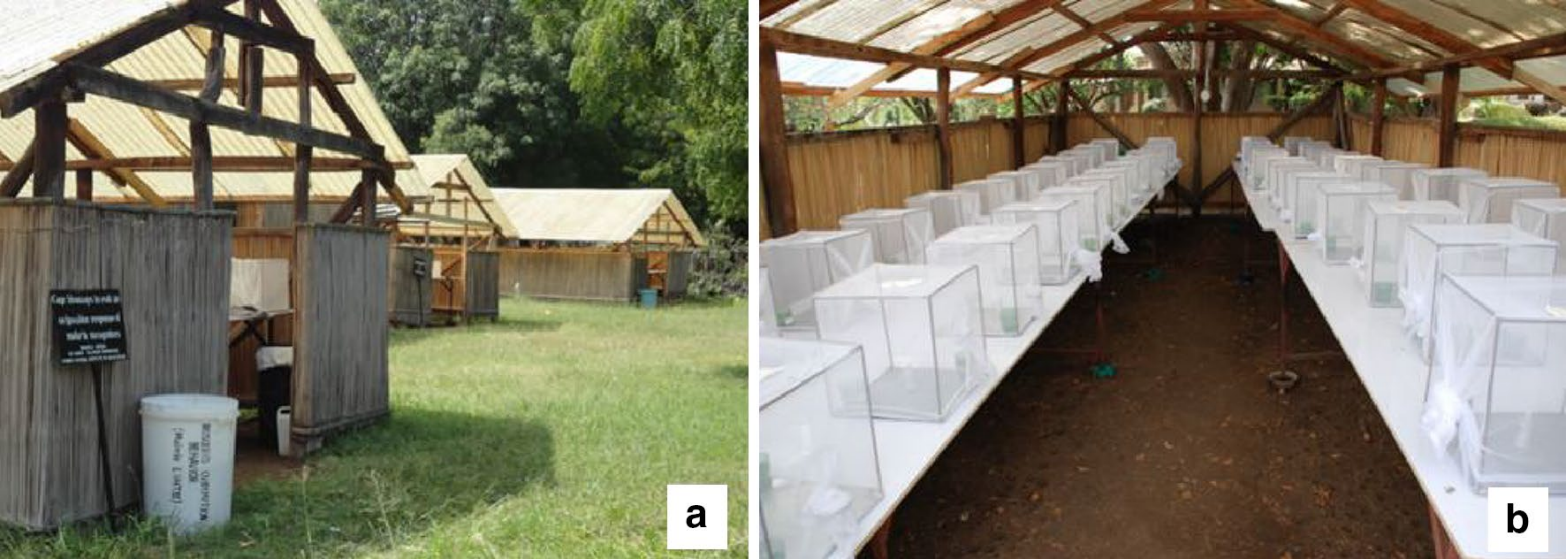
Experimental set-up of two-choice egg-count bioassays. **a** makeshift huts, **b** cages set up in hut at icipe-Thomas Odhiambo Campus, western Kenya.

Experiments were carried out at the International Centre of Insect Physiology and Ecology, Thomas Odhiambo Campus at Mbita on the shores of Lake Victoria, Western Kenya (0°26′ 06.19″ South; 34°12′ 53.12″ East; altitude 1,149 m). Choice egg-count bioassays were carried out in sheds, 10 m long × 5 m wide × 2.8 m high with walls constructed from dry reed mats and roofs of translucent corrugated polycarbonate sheets. Every shed contained two tables with capacity to hold 50 cages with a gap of 40 cm between each cage (Figure 1). Experiments were carried out at ambient conditions of temperature, humidity (mean daily temperature 27 ± 5°C, relative humidity 55 ± 10%) and light.

### Mosquito rearing procedures

Insectary-reared *An. gambiae* s.s. and *Anopheles arabiensis* (Mbita strains) were used for this study. Briefly, 2–3 days old mosquitoes were allowed to feed on a human arm for 15 min on two consecutive evenings at 19:00 hours. On the third day, blood-fed females were allowed to oviposit on wet filter papers provided overnight in the cage. Eggs were dispensed in 20-L plastic tubs (41 cm diameter, 8 cm deep) half-filled with non-chlorinated tap water purified by filtering through a charcoal-sand filter (hereafter called tap water). Hatched larvae were fed with ground Tetramin^®^ baby fish food (Tetra, Melle, Germany) twice daily. Pupae were collected into 10-cm diameter plastic cups filled with 200 ml of tap water and left overnight in mosquito cages for adults to emerge. Adults were maintained on 6% glucose ad libitum using absorbent paper wicks propped in 25-ml vials filled with glucose solution.

### Mosquito dissections

Females were immobilized by placing them in a refrigerator at 4°C for 15 min. Terminalia and the near terminal abdominal segment (segment IX) were severed in normal saline to expose spermathecae. Slide mounts of spermathecae were inspected using a microscope at 1,000× magnification for the presence of motile spermatozoa—a confirmation for insemination. The abdominal segments VII and VIII were gently severed to expose ovaries. The ovaries were observed at a magnification of 200× to evaluate stages of egg development. Mosquitoes with mature eggs, boat-shaped with fully developed floats, were categorized as gravid. To estimate the size of a female, the length of the left wing was measured from the axial incision to the tip (omitting the fringe setae) to the nearest 0.1 mm [43, 44].

### Cages and oviposition cups

Experiments were carried out in standard cages (30 × 30 × 30 cm) or in large cages (60 × 60 × 60 cm). The cages had a steel framework on a galvanized metallic base and covered with fine cotton mosquito netting. The cage net also had an insert sleeve for introducing and retrieving oviposition substrates and gravid mosquitoes. Oviposition substrates were offered in 7-cm diameter, 100-ml clear borosilicate crystallising glasses (Pyrex^®^, hereafter called oviposition cups). Prior to any experiment oviposition cups were autoclaved and kept at 200°C for 2 h to reduce the possibility of bacteria and odorant contamination. Individual gravid mosquitoes (indicated by an enlarged, pale white abdomen) were introduced into the cages and provided with either one or two oviposition cups containing 100 ml of tap water.

### Experimental procedures I: improving the egg laying success of *Anopheles gambiae* s.s. for egg-count experiments

It is important to produce large numbers of gravid mosquitoes that are consistent in their egg-laying behaviour during oviposition experiments. Here a series of experiments was carried out with caged local mosquito strains to evaluate and optimize major factors that are known to affect egg laying of mosquitoes in laboratory colonies including mating and insemination [45, 46], host source of blood meal [47–51] and the periods between blood feeding and the provision of suitable substrates [52–54].

#### Providing sufficient time for mating

Relatively young females are selected and blood fed when preparing gravid mosquitoes for experiments to maximize the proportion that survive until experiments are done 2–3 days after the blood meal. It was explored if young females, separated from males after 3 days, and then blood fed are sufficiently inseminated to develop and lay eggs 3 days later and whether the oviposition success can be improved by holding females with males after the blood meal. Two groups of 300 3-day old female *An. gambiae* s.s. mosquitoes were put in separate standard cages. These mosquitoes had spent the first 3 days of their adult life in colony cages with over 1,000 male and female conspecific mosquitoes. In one cage 300 sibling males of the same age were added, whilst in the other cage no male mosquitoes were included. Both groups of mosquitoes were then starved of sugar solution for up to 6 h. Tap water saturated cotton towels folded to a size of 50 × 25 cm were placed over the cages to maintain the relative humidity (RH) between 68 and 75%. The starved mosquitoes were permitted to blood feed from a human arm at twilight (between 18:30 and 19:30) for 15 min in imposed total darkness. Mosquitoes that were not fed after the first blood meal were removed from the cage, killed and discarded. Sugar solution was then replaced in the preparation cage until 12:00 the following day when the mosquitoes were starved again in preparation for a second blood meal the same evening. After blood feeding the mosquitoes were left in the insectary at temperatures that averaged 27 ± 2°C and were only retrieved 72 h later at the onset of experiments. The mosquitoes were 7 days old when egg-count cage experiments were implemented. Fifty females were selected based on their abdominal appearance from each of the two cages and transferred individually to standard cages. Each female was offered a single oviposition cup with 100 ml of tap water. The presence and number of eggs was recorded after 16 h (17:00–08:00). This experiment was carried out on three occasions (rounds; 3 × 50 individual females per treatment). Identical experiments were carried out with *An. arabiensis.*

#### Impact of cage size, mosquito age and body size on insemination

The previous experiment revealed a very low (<30%) oviposition rate in *An. arabiensis* even when kept with males for 7 days. Therefore, an experiment was designed to investigate the role of cage size, age and size of mosquitoes on the insemination success (proportion of inseminated females) of *An. gambiae* s.s. and *An. arabiensis*. Experiments were carried out in standard and large cages in parallel. For both species, 1,400 pupae were placed in plastic cups (10 cm diameter) filled with 200 ml of tap water positioned in each of two standard cages for 24 h. From that cage, 300 newly emerged male and 300 female mosquitoes of each species were transferred into separate cages of the two sizes. Six per cent glucose solution was provided in all cages ad libitum. After 3 days, 25 female mosquitoes were randomly selected from each cage by a technician unaware of the objectives of the study and dissected to evaluate insemination and to measure the wing lengths. The same number of females was dissected for days 4, 5 and 6. The experiment was implemented for three rounds with different batches of mosquitoes. Mosquitoes in this experiment were not offered blood meals.

#### Optimal timing of cage experiments

To have a consistently large proportion of females respond in oviposition experiments, it was important to establish the optimum interval between the last blood meal and the bioassay for the local mosquito strains. Furthermore, putative test substrates for oviposition in mosquitoes (e.g., bacteria solutions, plant infusions, volatile inorganic compounds) are often unstable. It is therefore important to target the experiments just before the peak in egg laying. Cage experiments were carried out with different mosquitoes 48 and 72 h after their second blood meal. ‘Gravid’ mosquitoes for experiments were prepared following standard procedures. For each experiment, 100 *An. gambiae* s.s. mosquitoes were individually offered two oviposition cups with tap water in two-choice egg-count experiments at 17:00. In 50 of the 100 cages, both oviposition cups were retrieved and replaced with two new cups containing tap water at 21:30, the remaining 50 cages were left undisturbed through the night. The aim here was to investigate if the caged *An. gambiae* s.s. have several oviposition peaks during the night and to explore when skip oviposition occurs. Half the cages remained undisturbed as a control to investigate if the exchange of cups might interfere with the oviposition response during the night. The experiment was ended at 08:00 the following morning and the number of eggs in each cup recorded. Both experiments were carried out for three rounds.

#### Understanding the impact of host sources of blood meal on egg laying

*Anopheles gambiae* s.s. is highly anthropophagic [55] and there is evidence that different host sources of blood meals have an impact on the oviposition rate and fecundity [51] of this species. An experiment was designed to elucidate the impact of feeding caged *An. gambiae* s.s. on non-human hosts on the proportion of females becoming gravid and the number of eggs laid by each female. Different groups of mosquitoes were blood-fed on either a human arm or rabbit. Blood meals on human arm were offered as described in the previous experiment. For rabbit host blood meals, fur was shaved on the ventral side of the rabbit in an area of 15 × 5 cm (approximately equal to the area exposed by an extended human arm covered with a latex glove). The rabbit was then held in a restrainer that limited movement and exposed the shaven underside. Mosquitoes to be fed were held in a cage positioned at the base of the restrainer allowing free access to the shaven area. In each treatment a group of 300 females were fed on two consecutive days. All mosquitoes that did not blood feed on the first day were removed from the cages. The blood-fed mosquitoes were then held together with 300 males in standard cages for 72 h. A total of 100 female mosquitoes were randomly selected from each of the two cages, aspirated out by a technician unaware of the objectives of the research, and dissected to determine if they were gravid or not. Another 25 females visually appearing gravid were purposively selected by an experienced technician from each of the two cages. These 25 females were tested individually in no-choice egg-count cage experiments to compare the proportion of females that laid eggs and the number of eggs laid per female fed on either rabbit or human blood. The experiment was done for three rounds using different batches of mosquitoes.

### Experimental procedures II: improving the experimental design of cage egg-count bioassays with *Anopheles gambiae* s.s.

To promote an empirical evaluation of substrate preferences using two-choice egg-count bioassays it is important to understand the natural egg-laying pattern of *An. gambiae* s.s. and take it into account when designing experiments. This experiment was aimed at identifying an appropriate design for egg-count oviposition studies with *An. gambiae* s.s. and at highlighting the importance of sample size. Specifically (1) the number of eggs laid by individual *An. gambiae* s.s. mosquitoes was estimated and their statistical distributions and variances explored; (2) skip-oviposition within experimental cages was quantified; and, (3) the variability in egg counts and response towards two equal choices of oviposition substrate analysed between rounds.

#### Two equal choice egg-count bioassays with individual gravid females to explore egg distribution and variability in egg-counts

**Figure 2:**
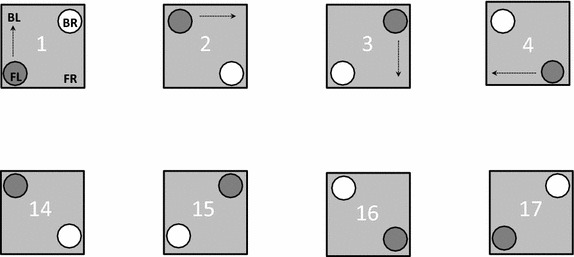
Illustration of the arrangement of oviposition cups and cages in two choice egg-count bioassays. The *solid circles* represent test cups which are arranged in the clockwise direction. Control cups (*open*) are positioned diagonally opposite. *FL* front right, *BL* back left, *BR* back right, *FR* front right.

Gravid females were prepared in standard cages as outlined above using 300 two to 3-day old female and 300 male *An. gambiae* s.s. of the same age. Mosquitoes were starved of sugar solution for up to 6 h before they were permitted to blood feed from a human arm at 18:30 for 15 min on two consecutive evenings. Mosquitoes that were not fully engorged with blood after the first blood meal were removed from the cage. Individual 6- to 7-day old gravid females were presented with two equal choices of tap water for oviposition. In order to prevent any possible bias associated with the position of the cup, the placement of cups was systematically varied between adjacent cages. The four corners of every cage were named relative to the front of the cage (inset-sleeve end) as front left (FL), back left (BL), back right (BR) and front right (FR). The first cup was placed at the FL position of the first cage and randomly referenced as ‘control’ or ‘test’. ‘Test’ cups in subsequent cages were moved one corner step in a clockwise direction (Figure 2). The second cups were added in the diagonally opposite corner and referenced as ‘control’. The egg-laying response of a gravid female towards these test and control cups was recorded as binary data. The numbers of eggs laid by every female in each cup was also noted. In total 41 rounds of two-choice egg-count experiments with different batches of mosquitoes were implemented. Between 20 and 50 individual female mosquitoes were exposed to the two equal choices per round but only 85–92% of all exposed females responded (laid eggs) per round (n = 17–46). The response of 1,443 females were analysed in total.

#### Sample size considerations

When implementing two-choice bioassays with two different oviposition substrates the assumption is that there is no preference between the two substrates. However, it is likely that the chances of a type 1 error (i.e., artefact preference for one substrate over then other) are increased with a small number of replicates. Therefore the data were used to estimate the sample size required for routine bioassays using power calculation for two-sample comparisons of proportions (power.prop.test function in R software) and for a single proportion compared to a known proportion [56]. A 50% distribution was assumed when equal choices are presented (p1 = 0.5). Power estimates were generated to predict a 15% increase or decrease (p2 = 0.65) in oviposition response to a test medium at sample sizes between five and 225 and generated estimates of effect sizes generated that can be detected with 80% power at a 5% significance level for the same range of sample sizes.

### Data analyses

Multivariable analyses were implemented using generalized estimation equations (GEE) to analyse how proportions were affected by test variables. Different batches of mosquitoes from different rounds of an experiment were considered clustered (not independent) and included in the GEE model as repeated measure. To evaluate the impact of including male mosquitoes in cages with blood-fed females on the proportions of mosquitoes that lay eggs, a GEE model was fitted with binomial distribution, logit link function and exchangeable correlation matrix. The presence of male mosquitoes (coded a 1 if a male is present, and 0 otherwise) in the holding cages was included in the model as a fixed factor. The association between the proportion of female mosquitoes inseminated and cage size (standard = 0, large = 1), mosquito age (a four-level categorical variable coded 3, 4, 5, and 6 days) and mosquito size (measured in terms of wing length) was assessed using two separate GEE models (binomial distribution, logit link function, exchangeable correlation matrix) for the two test species. An interaction term of age and cage size was also included in the model. Similar models were fitted for the experiment on host blood meal sources with the blood meal source as a fixed factor, and the experiment on oviposition time with time period as a fixed factor. The mean numbers of eggs and their corresponding 95% confidence intervals (CIs) for two treatments were calculated as the exponent of the parameter estimates based on generalized linear models with negative binomial distributions with no intercept included.

Egg numbers laid by individual females were tested for normality using the Kolmogorov–Smirnov test. Additionally, overdispersion (i.e., meaning the variability in the data is not equal to the mean, as in the Poisson distribution) was assessed by inspecting the residual deviance, which follows a Chi-squared distribution where the expected value should be close to the degrees of freedom if the data are not overdispersed. Overdispersion is a problem because it may cause standard errors of the estimates to be deflated or underestimated, i.e., a variable may appear to be significant when in fact it is not. The assumption of homogeneity of variance in the correlated count data collected from control and test cups was tested with the Pitman–Morgan test [57].

In the two equal choice egg-count bioassay one mosquito were presented with two cups with tap water for oviposition. The data derived from these two cups per individual mosquito were related and therefore a mosquito was considered a cluster in the GEE models. GEE models assuming exchangeable working correlation and with a negative binomial distribution with a log link function fitted were used to explore differences in egg counts between control and test cups and between rounds (fixed factors), whilst GEE models with a binomial distribution and logit link faction fitted were used to estimate the odds of a female choosing the test cup over the control. All mean counts or mean proportions per treatment and their 95% CIs were calculated as the exponential of the parameter estimates for models with no intercept included.

Data were analysed with IBM SPSS Statistics Version 20 [58] and R software version 2.13.2 using various functions from the packages MASS, epicalc, lme4, effects, geepack, aod and gee [59].

### Ethical considerations

Ethical approval for this study was obtained from the Kenya Medical Research Institute’s Ethical Review Committee (Protocol no. 422).

## Results

### Including males in holding cages after blood meals increases the proportion of ovipositing females

The odds of a female *An. gambiae* s.s. laying eggs were nine times greater if, after a blood meal, she was held with males than without them (OR = 9.0, 95% CI 7.9–9.5, p < 0.01). On average 84% (95% CI 77–89%) of females laid eggs when held with males compared to 36% (95% CI 29–44%) when held without. Whilst the total number of eggs laid by females held with males [2,904 eggs (95% CI 2,844–2,968)] was three times as high as the total number laid by females kept separated from males after blood-meals [994 eggs (95% CI 959–1,030)], the mean number of eggs laid per female was similar in mixed-sex cages [66 eggs, 95 % CI 44–99 eggs] and female only cages (54 eggs, 95% CI 36–82) highlighting the benefit of observing individual rather than groups of mosquitoes.

Only 25% of *An. arabiensis* (95% CI 15–41%) laid eggs. The likelihood of laying eggs was not associated with the presence or absence of males in the cages after blood feeding (OR 1.92, 95% CI 0.62–5.98, p = 0.658) and dissections showed that a large proportion (>50%) of females that did not lay eggs were not inseminated. The mean number of eggs laid per female that laid was 63 (95% CI 59–68).

### Age and body size can impact on insemination success in *Anopheles gambiae* and *Anopheles arabiensis* irrespective of cage size

**Figure 3:**
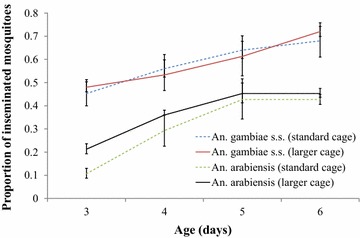
Insemination rates of caged *Anopheles gambiae* s.s. and *Anopheles arabiensis* (Mbita strains) in standard (30 × 30 × 30 cm) and large (60 × 60 × 60 cm) cages with increasing age of the females.

The proportion of inseminated mosquitoes increased with time and age for both species (Table 1; Figure 3). However, the overall odds for *An. arabiensis* were only 0.16 (95% CI 0.12–0.23, p < 0.01) compared to *An. gambiae* s.s. The mean proportion of inseminated *An. gambiae* s.s. increased linearly to 72% (95% CI 61–81%) 6 days after emergence. The insemination rate of *An. arabiensis* peaked 5 days after emergence with 45% (95 CI 36–57%) inseminated (Figure 3). Cage size did not improve insemination rate for *An. gambiae* s.s.. In *An. arabiensis*, an improved insemination rate was observed in larger cages for 3-day old females but not for older females (Table 1). The average length of the left wing of *An. arabiensis* was 4.20 mm (95% CI 4.16–4.23 mm) compared to 3.76 mm (95% CI 3.70–3.82 mm) for *An. gambiae* s.s. While body size did not affect *An. gambiae* s.s. insemination, *An. arabiensis* females were 6.6 times more likely to be inseminated with every unit increase in wing length (Table 1).

**Table 1:** Multivariable analysis of factors tested in association with the rate of insemination

Variable	*Anopheles gambiae* s.s.		*Anopheles arabiensis*	
	OR (95% CI)	P value	OR (95% CI)	P value
Age of mosquito in days				
3	1		1	
4	1.34 (1.30–1.48)	<0.001	3.40 (2.40–4.74)	<0.001
5	1.98 (1.62–2.43)	<0.001	6.23 (3.23–12.0)	<0.001
6	2.81 (1.75–4.52)	<0.001	6.20 (3.99–9.64)	<0.001
Cage size				
Standard	1		1	
Large	1.02 (0.97–1.07)	0.457	2.13 (1.66–2.73)	<0.001
Body size				
Wing length	0.68 (0.33–1.37)	0.278	6.68 (2.57–17.4)	<0.001
Interaction between mosquito age and cage size				
3*standard	1		1	
3*large	1		1	
4*standard	1		1	
4*large	0.81 (0.63–1.04)	0.099	0.61 (0.32–0.69)	<0.001
5*standard	1		1	
5*large	0.80 (0.42–1.51)	0.493	0.53 (0.37–0.75)	<0.001
6*standard	1		1	
6*large	1.06 (0.67–1.70)	0.799	0.47 (0.42–0.52)	<0.001

*OR* Odds ratio, *CI* confidence interval.

* Statistical interaction.

### Egg-count cage bioassays are best implemented 72 h after the last blood meal during the peak oviposition time between 17:00 and 21:30

Due to the poor oviposition success in the colony-reared *An. arabiensis* the following experiments were implemented with *An. gambiae* s.s. only. Female *An. gambiae* s.s. were 8.7 times (95% CI 4.3–18.4 times, p = 0.290) more likely to lay eggs when provided with substrates 72 h after blood meals compared with females provided with substrate after 48 h. On average 81% (95% CI 71–93%) of females presented with oviposition substrate 72 h after blood meals laid eggs compared to only 33% (95% CI 32–35%) 48 h after blood meals (Table 2). Approximately 76% (95% CI 71–82) of females laid eggs whether cups were left untouched over night or changed by 21.30, suggesting that changing the cups did not interfere with oviposition. About 96% of females that laid eggs in the experiment where cups were exchanged did so between 17:00 and 21:30 (114/119). The tendency for individual female mosquitoes to lay eggs in both cups (also known as skip oviposition) was observed in 26% (95% CI 19–30) of all responding females and 90% (95% CI 89–96%) if it took place before 21:30. Only two mosquitoes laid eggs before and after 21:30 (Table 2) and one only after 21:30.

**Table 2:** Evaluation of egg laying periodicity in caged *Anopheles gambiae* s.s. (Mbita strain)

	N (exposed)	n (responded)	Percentage of mosquitoes that laid eggs (95% CI)	P value
Duration after blood meal (cups left overnight) (h)				
48	200	75	33 (32–35)	<0.001
72	150	122	81 (71–93)	
Egg laying period (72 h after blood meal)				
17:00–21:30	150	114	96 (94–100)	<0.001
21:30–08:00		3	3 (2–5)	
Both periods		2	2 (1–4)	
Skip oviposition (72 h after blood meal)				
17:00–21:30	31	28	90 (89–96)	<0.001
21:30–08:00		1	3 (2–5)	
Both periods		2	6 (5–7)	

### Blood meal sources from a secondary host reduces the proportion of females that become gravid but does not affect egg numbers laid by gravid females

Females fed on rabbit blood were less likely to become gravid compared to those fed on human blood (Table 3). When selected from the cage randomly, on average 59% (95% CI 44–73) of those females offered blood from a rabbit were gravid and laid eggs while 83% (95% CI 68–92%) of females fed on a human blood were gravid and laid eggs. Of those carefully selected as gravid based on their abdominal appearance, equal proportions of females from both treatments laid eggs when offered an oviposition medium. The mean number of eggs laid by individual gravid females also did not depend on the host source of blood meals (Table 3).

**Table 3:** Effect of host source of blood meal on oviposition of caged *Anopheles gambiae* s.s. (Mbita strain)

	Mean (95% CI)	OR (95% CI)	P value
Percentage of blood-fed mosquitoes gravid at dissection			
Human arm	83 (68–92)	1	
Rabbit	59 (44–73)	0.30 (0.14–0.66)	0.030
Percentage of gravid mosquitoes that laid eggs in cage experiments			
Human arm	72 (57–83)	1	
Rabbit	73 (59–84)	1.07 (0.95–1.20)	0.852
Number of eggs per gravid female that laid			
Human arm	64.0 (57.1–71.8)	1	
Rabbit	62.1 (52.2–73.9)	0.97 (0.85–1.11)	0.661

*OR* Odds ratio, *CI* confidence interval.

### Individual *Anopheles gambiae* s.s. lay a highly variable number of eggs despite standardized preparation procedures

**Figure 4:**
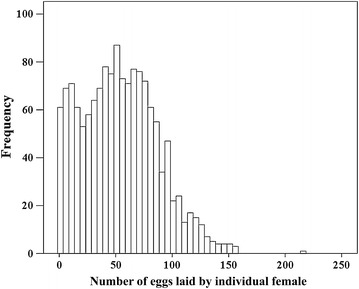
Histogram showing the frequency distribution of egg counts from 1,443 individual *Anopheles gambiae* s.s. (Mbita strain) females.

The total number of eggs laid by each of 1,443 mosquitoes individually provided with two cups of tap water was highly variable and ranged between one and 214 eggs (interquartile range 48), with a median of 52 eggs. Egg numbers were overdispersed with the variance exceeding the mean indicating an overdispersed distribution (Figure 4).

### The response of gravid females presented with two equal choices can be skewed when egg-counts are compared

In total 77,664 eggs were laid by 1,443 mosquitoes tested individually over 41 rounds; 41,113 (53%) eggs were laid in cups randomly labelled as test cups, and 36,551 (47%) in control cups. In addition to the overdispersed distribution of eggs, the two correlated variances of the egg counts in control and test cups were not homogeneous (p < 0.01). Generalized linear modelling with a negative binomial distribution fitted indicated that the differences in egg counts between control and test cups were small but statistically significant [rate ratio (RR) 1.13 (95% CI 1.01–1.25, p < 0.01)]. Furthermore, counts differed significantly between rounds (p < 0.001) with mean number of eggs laid per female in a cup in different rounds ranging between 17 (95% CI 13–20) and 46 (95% CI 39–55).

### Comparing the proportional distribution of eggs leads to more reliable inference than using absolute egg counts

**Figure 5:**
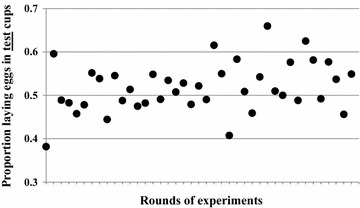
Proportion of responses (presence of eggs) received by the test cups in two equal choice tests out of the total responses (test cups + control cups) counted per experimental round (n per round = 15–43).

Rather than evaluating the actual egg counts, the proportion of eggs laid in test *versus* control cups (experiments with groups and individuals) or the proportion of females (experiments with individuals) selecting test *versus* control cups for oviposition can be compared. A total of 1,902 cups (out of 2 × 1,443 = 2,886 cups) received eggs; 979 test cups (51%) and 923 (49%) control cups in the 41 rounds of experiments. The distribution of individual responses towards two equal choices was therefore more balanced than the comparison of egg numbers (see above). Consequently, generalized linear modelling with a binomial distribution fitted showed that that the odds of a female choosing one cup over the other when both contain the same oviposition substrate was similar (p = 0.08) with a mean proportion of 51.4% (95% CI 49.0–53.8%) selecting the test cup for oviposition. This relatively balanced outcome is based on a very large number of samples. Looking at the individual rounds containing between 17 and 45 samples only (Figure 5), the proportions of females selecting the test cup is highly variable with significant between-round differences (p < 0.01). This natural baseline variability must be measured during experiments and taken into account when implementing choice experiments with different substrates. Otherwise, it would easily lead to false inferences especially where sample sizes are small.

### High between-cage variability in egg-counts and proportions must be expected when testing small groups of gravid females in egg-count cage bioassays

**Figure 6:**
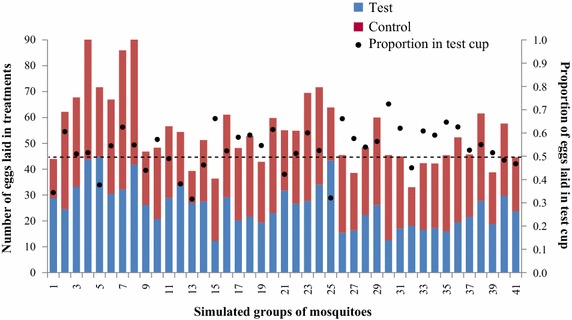
Mean number of eggs per female laid in test and control cup and proportion of eggs laid in test cup. Analysis based on 41 simulated groups of mosquitoes.

**Figure 7:**
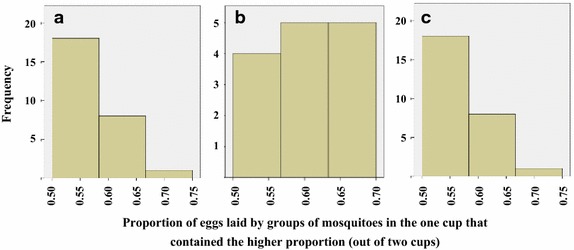
Frequency distribution of the proportions of eggs laid in one cup (higher proportion) over the other in two equal choice tests for simulated groups. **a** all 41 groups, **b** groups with ≤30 individuals, **c** groups with >30 individuals.

The majority of choice egg-count bioassays published for *An. gambiae* s.l. have been implemented with groups of mosquitoes [34, 35, 60–63]. This scenario was simulated by combining the egg-counts for test and control cups of all individual mosquitoes tested (responders) in a round. Therefore, the hypothesised group sizes varied from 17 to 46 mosquitoes per cage. Conventionally, the number of eggs laid per female is estimated by dividing the total number of eggs counted by the number of females introduced in the cage. Note that in contrast to the simulation under these experimental conditions, investigators cannot be sure of the actual number of females that laid and based on here presented observations it must be assumed that approximately 20% of the introduced females do not lay even when prepared under optimal procedures. Figure 6 shows the number of eggs per female in the test *versus* the control cups for the 41 simulated groups (replicates), including the proportion of eggs laid in the test cup per group. The mean number of eggs per female per group was highly variable and ranged from 33 to 92 between the assumed replicate cages. Similarly, the proportional distribution of eggs between the two cups, containing the same oviposition substrate, was in nearly half the groups unequal with one cup having >60% of all eggs laid (Figure 7a). Notably, there was a negative correlation between the number of females per cage and the difference in proportion of eggs laid in test *versus* control cups (Spearmans rho = −0.35, p = 0.03). If a group consisted of less than 30 responders an unbalanced distribution of eggs (>60% of eggs in one cup) between the two equal choices occurred twice as often as a balanced one (Figure 7b), whilst in groups with more than 30 responders an unbalanced distribution was less frequent (Figure 7c).

### One-third of gravid *Anopheles gambiae* s.s. distribute their eggs in more than one oviposition medium (‘skip-oviposition’)

**Figure 8:**
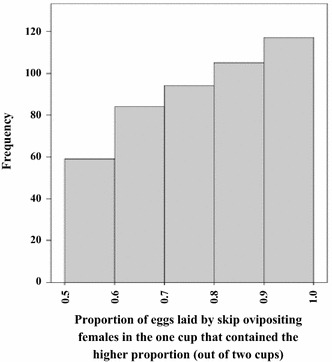
Frequency distribution of the higher proportion (>0.5) of eggs laid in one cup by skip-ovipositing females.

Individual *An. gambiae* s.s. females did not always make mutually exclusive choices of cups to lay their eggs when provided with two substrates. Of the 1,443 responders, 32% (459) laid eggs in both cups provided in the cage. Nevertheless, significant variability (p < 0.01) was observed between batches of mosquitoes (rounds), with the proportion of skip oviposition ranging between 17 and 61% in individual experimental rounds. On average 32.4% (95% CI 29.0–35.8%) of the females per round laid eggs in both cups presented. Females that skip-oviposited did not lay more eggs compared to those that laid all eggs in one substrate (p = 0.873). Importantly, most females that laid in both cups did not distribute their eggs equally in the identical substrates. The unequal distribution of eggs can therefore be wrongly interpreted as a preference for the substrate that received the higher number of eggs. In most cases a larger egg batch was laid in one cup and a smaller batch in the other cup (Figure 8). Three-quarters of the females that skip-oviposited laid two-thirds or more in one and one-third or less of their eggs in the other cup.

**Figure 9:**
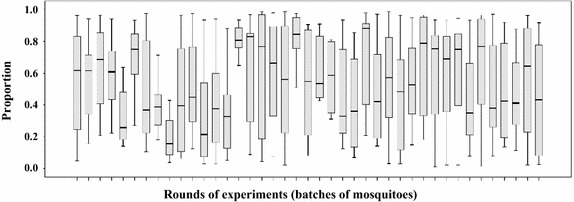
Median proportion of eggs laid in the test cups by skip-ovipositing females (n = 4–20) in every experimental round.

The unequal egg distribution might contribute to skewed egg counts, especially when the number of individuals tested in a sample and/or the number of replicate samples are low. This is illustrated by Figure 9 where the median proportion of eggs laid in the test cups is shown for every experimental round. Rarely were the proportions of eggs laid by skip-ovipositing females (n = 4–20) equally distributed in a single round. Nevertheless, on average for all 459 skip-ovipositing females, 54% (95% CI 45–63%) of the eggs were laid in test cups emphasizing the importance of a large enough sample size.

### To detect an increase in oviposition response of 15% as compared to the baseline proportion (80% power and 5% significance) at least 165 responders need to be tested in each treatment group

**Figure 10:**
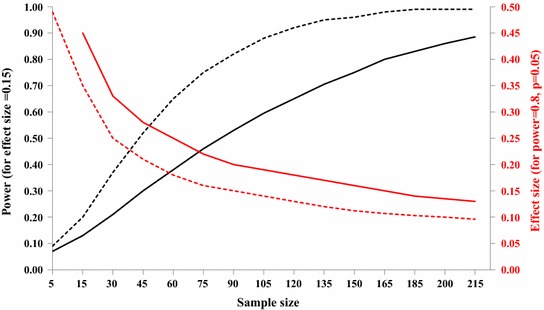
Description of the measurable powers (*black*) and effect sizes (*red*) of tests with different sample sizes (number of mosquitoes) for two proportions at the 0.05 significance level. *Solid lines* sample size considerations based on power calculation for two-sample comparisons of proportions. *Dashed line* sample size calculation for the inference for a single proportion comparing to a known proportion (0.5) suitable for testing large groups where this baseline proportion can be confirmed.

Based on the design considerations presented above, when implementing egg-count cage bioassays it is suggested to statistically compare two proportions derived from two independent (separate) random samples. The null hypothesis H_0_ is that the two samples’ proportions are the same. The notation for the null hypothesis is H_0_: p_1_ = p_2_, where p_1_ is the baseline proportion from choice experiments with two equal choices (control substrate *versus* control substrate), and p_2_ is the proportion from the experimental test comparing a putative oviposition cue against a control. The sample size will depend on the effect size one wants to detect. Here it was chosen to simulate (1) the relationship between sample size and the power of a study at 5% significance level at an effect size of 15% increase of p_2_ as compared to p_1_ and, (2) the relationship between sample size and effect size (p_2_) at a fixed power of 80% at 5% significance level (Figure 10).

Based on sample size calculations for two independent proportions, 165 responders need to be tested in each group (165 for p_1_ and 165 for p_2_; total 340) to detect an increase or decrease in oviposition response of 15% (p_2_ = 0.65) compared to the baseline proportion (p_1_ = 0.50) at 80% power and 5% significance. With a smaller sample size the effect size that can be detected increases, i.e., 90 replicates in each treatment arm can detect a difference between the proportions of not less than 20% (p_1_ = 0.50 and p_2_ = 0.70) and 30 replicates of not less than 33% (p_1_ = 0.50 and p_2_ = 0.83) (Figure 10).

These sample size considerations apply irrespective of whether the proportions of eggs laid by groups of mosquitoes per cage or by individual mosquitoes per cage are compared since in both cases only a single data point per cage can be recorded and the proportion of non-responders in the cage is unknown. Nevertheless, if large groups (>30 responders per cage for example) are used where the baseline proportion can be predicted to be close to 50% with some certainty it might be justifiable to use the sample size calculation for the inference for a single proportion comparing to a known proportion (0.5). In this case 85 replicate cages would be required for detecting a 15% increase compared to the baseline proportion at 80% power and 5% significance (Figure 10). Whilst this number of replication appears to be considerably lower it needs to be observed that over seven times more gravid females would be required in this experimental design (85 × 30 = 2,550) than when using individual females and two treatment arms (2 × 165 = 330).

## Discussion

### Improving the egg-laying success of gravid females for egg-count experiments

**Figure 11:**
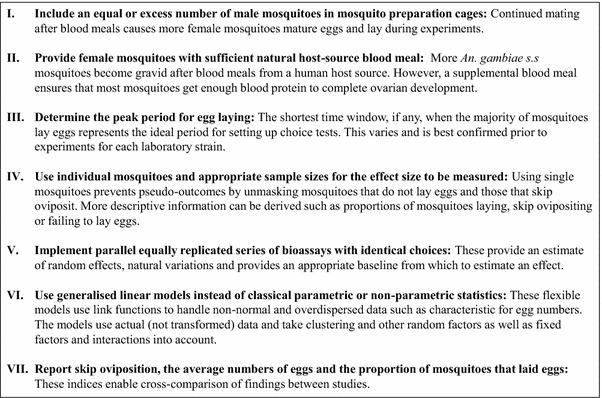
Summary recommendations for implementing two-choice cage egg-count bioassays for evaluating oviposition substrate preferences of malaria vectors.

To implement empirical egg-count experiments with replicable and generalizable results it is vital to ensure a consistent and predictable oviposition rate in the test mosquitoes. This study highlighted important considerations (summarized in Figure 11) when preparing gravid *An. gambiae* s.s. for two-choice egg count experiments: the insemination rate of the test mosquitoes, the blood meal host source and the timing and duration of the actual experiments.

Consistent with previous studies, insemination was shown to be important for egg laying by *An. gambiae* s.l. [45, 46]. The proportion of test females that laid eggs in the bioassays more than doubled when they were held in cages with males after blood feeding providing a longer period to mate. This gives further evidence that at least in laboratory settings mating in this species continues after the females have taken a blood meal. Depending on the age of insects at blood feeding this could be of great consequence. Cages with blood-fed females must be conditioned for insemination by including male mosquitoes especially when test females are blood fed at a relatively young age (here 2–3 days). In experiments evaluating the rate of insemination with non-blood-fed mosquitoes it was shown that approximately one-fifth of *An. gambiae* s.s. were still virgins when 6 days old (the average age of test mosquitoes across studies). This might explain the similar proportion of test mosquitoes that failed to lay eggs in the bioassays even under optimized preparation procedures. Increasing the number of males in cages might improve both the rates of insemination and egg laying [64]. However, Verhoek and Takken [65] have demonstrated that ratios of 3:1 male to female do not significantly improve the rate of mating over a 1:1 ratio for *An. gambiae* s.l.

Rabbit blood meals resulted in a lower proportion of mosquitoes that eventually became gravid. This suggests that the common practice of substituting human hosts with rabbits, and possibly other secondary host sources of blood [63, 66, 67] potentially reduces the number of gravid mosquitoes and therefore increases the risk of including mosquitoes that will not lay eggs in bioassays. Excluding mosquitoes that did not lay eggs from the analysis showed that the actual mean number of eggs laid per female that became gravid after the blood meal was the same irrespective of the source of blood. If groups had been tested instead a false lower mean numbers of eggs with rabbit blood meals would have been inferred. Great caution is advised in selecting gravid mosquitoes where secondary host sources of blood are used in preparing test mosquitoes. By using individuals it is possible to implement choice test even where the impact of the host-source of blood meal is large or unknown. Mosquitoes that do not lay eggs can be removed from the final data set and reported as a separate entity of interest.

McCrae [53] wrote “For any study of oviposition to be complete it would be valuable to know the probable time of its occurrence as a basic guide for laboratory procedures”. The vast majority of the Mbita strain of *An. gambiae* s.s. did not yet lay eggs 48 h after the last blood meals; egg laying was constrained to early evening hours of the third night (≈72 h) after blood meals. This confirmed the findings of Haddow and others [68]. Consequently, egg-count cage bioassays with the Mbita strain are best done between 17:00 and 21:30 on the third night after the last blood meal. However, controversial results have been published in the past. Other studies with *An. gambiae* s.s. have shown that some strains are laying eggs 48 h after a blood meal and it was suggested that egg-laying times depend on local conditions, blood-feeding times and temperature [53]. Some studies also show that *An. gambiae* s.s. can lay eggs at any time throughout the dark phase [53, 54]. In consideration of these divergent findings, it is strongly recommended that oviposition periodicity studies precede all oviposition studies with different strains of this species. This does not only apply to egg-count cage bioassays but is equally important when investigating chemoreception in gravid females and changing sensilla sensitivity in response to changes to the physiological stage of a mosquito. These studies are often done 24 and 48 h after a blood meal [30, 69], which might not necessarily coincide with the time a female searches for an oviposition site.

The insectary-reared Mbita strain of *An. arabiensis* showed low rates of insemination compared to *An. gambiae* s.s. from the same area. At best 45% of all female *An. arabiensis* mosquitoes were inseminated after 6 days when held with an equal number of males throughout. There is some evidence that *An. arabiensis* is more difficult to mate and colonize in the laboratory compared to *An. gambiae* s.s. [70], although others have shown contrasting results where the rate of insemination in *An. arabiensis* of every age between 1 and 7 days was higher than that of *An. gambiae* s.s. [65]. Their findings were probably due to longer colonization of the strain which selected for this trait. Increasing the size of holding cages to increase mating activity and insemination success in *An. arabiensis* was without significant gain. Low insemination and consequently low oviposition rates make it difficult to study the oviposition response of *An. arabiensis* to different oviposition substrates. Especially, when groups of *An. arabiensis* are used, caution should be exercised in interpreting the results by examining the mean egg numbers critically to ensure that the majority of the exposed females actually laid eggs. It has been shown that larger females were more likely to be inseminated compared to smaller ones. Attempting to optimize larval rearing conditions to increase adult body size and selecting for the largest females from the colony cages for experiments might thus be a reasonable approach to increasing oviposition rates in egg-count cage bioassays.

### Improving the experimental design of cage egg-count bioassays with *Anopheles gambiae* s.s.

Using two equal choice egg-count bioassays with individual gravid mosquitoes illustrated the importance of (1) suitable experimental design based on the behavioural ecology of *An. gambiae* s.s.; (2) estimated sample sizes; and, (3) appropriate statistical analyses (Figure 11). This study confirmed that egg counts of individual female *An. gambiae* s.s. of the same age fed on the same source of blood and reared under standardized conditions are highly variable and overdispersed. Lyimo and Takken [71] previously demonstrated that individual newly emerged *An. gambiae* s.l. of the Muheza strain laid between 48 and 178 (mean 111) eggs while wild field populations laid an equally variable 66–290 (average 150) eggs. Hogg and Hurd [72] later confirmed variations in egg numbers showing that wild *An. gambiae* s.s. and *An. arabiensis* of Gambia laid between 20 and 180 eggs and five and 160 eggs, respectively. These wide disparities in egg numbers of individual females have also been shown for laboratory strains of other Anophelinae including: *Anopheles stephensi* [73], *Anopheles sergenti* [74]*, Anopheles multicolor,* and *Anopheles pharoensis* [75]. Suleman and others [73] noted that a small portion of *An. stephensi* females laid a very high number of eggs per batch, leading to a negative binomial distribution as also well demonstrated for *An. gambiae* s.s. in this study. Similar heterogeneity in egg numbers between individual females have also been shown for *Aedes aegypti* [76]. This may be a general trait of mosquitoes that lay single eggs, rendering the use of egg numbers to gauge oviposition substrate preferences inappropriate especially with small groups of mosquitoes [37]. It was demonstrated that the high variation in the number of eggs laid by individual females can lead to an unequal distribution of eggs in equal substrates. This disproportion persisted even with very large sample size.

Exploring the pattern of ‘skip oviposition’ in *An. gambiae* s.s., it was demonstrated that approximately one-third of all gravid *An. gambiae* s.s. distribute their eggs in more than one oviposition site, a behaviour that is well known in *Aedes* mosquitoes [77, 78], but has been poorly described in *An. gambiae* s.l. species [36, 79] in laboratory egg-count experiments, possibly because most experimenters use groups of mosquitoes, which masks skip oviposition. There is also indirect evidence of skip oviposition from one study in the field [80] showing that this is not an artefact trait of colonized mosquitoes but rather an inherent trait of the species. Skip oviposition represents a response of the gravid female to the substrates and should not be excluded from analyses. Skip ovipositing females choose to use both substrates, therefore not rejecting any, an important event with reference to comparative preference of substrates. Importantly, *An. gambiae* s.s. females do not distribute their eggs in equal proportions but in most cases lay two-thirds in one and one-third in the other oviposition cup. Since observations in this study are based on equal choices, it is clear that the higher egg batch does not indicate a preference. It is important to note that individual skip ovipositing female did not lay more eggs compared to those individual females that laid in a single cup.

In experiments, where groups of females are analysed in oviposition assays, the marked heterogeneity of egg numbers laid by individual females combined with skip oviposition is likely to increase the variance in the system and this could lead to a type 1 error where an unequal distribution of eggs between the test and control solutions is wrongly considered to be true, especially if group sizes are small. Here it was illustrated that this frequently happens when group numbers per cage are below 30 responders. Considering that of those, probably a fifth or more mosquitoes do not lay eggs, a skewed distribution can be expected and only a large number of cages can be able to detect true differences of substrates. Since many choice experiments with anophelines are done with groups much lower than 30 [22, 30, 35, 61], results need to be interpreted with caution.

This study demonstrated that observing individual mosquito’s responses to oviposition substrates rather than groups has a number of advantages. This approach ensures that only responders are included in the data analysis. It allows the analysis of choice based on a binary outcome, the enumeration of egg numbers of individual females and the observation of skip oviposition, which has previously been shown to be influenced by the suitability of a substrate [36]. Last but not least, the necessary number of replications can be achieved with a smaller number of gravid females compared to when groups are used.

Sample size considerations are rarely reported for entomological studies and the number of replications hardly ever justified in publications. This study illustrates that insufficient replication might not only hamper the ability to show a significant effect due to the lack of power, but also demonstrates that a small number of replicates and small group sizes can result in significant artefact differences in oviposition responses in two-choice experiments purely based on stochastic effects rather than due to a treatment effect. Misinterpretation of results can be reduced by sufficient replication and validation of the experiment by implementing a control experiment preferably in parallel [81].

The underlying hypothesis of a choice experiment is that when two (or more) equal choices are presented the response towards these choices is equally proportional with odds of success of 1:1 (baseline or control). Choices by virtue of the design of the experiment should be analysed as proportions rather than absolute counts, especially when count data are highly variable. If an oviposition cue is presented that is either preferred or avoided by gravid females a significant diversion from the baseline is expected. It was shown that there is a high variability in the response towards a test and control cup containing the same substrate in individual rounds of experiments highlighting the importance of large sample sizes and the implementation of an experiment over several rounds with different batches of mosquitoes. The behaviour of mosquitoes from the same batch might be affected for example by their rearing history and/or by the climatic conditions during the experiment or other non-measurable random effects. Replicate tests with mosquitoes from the same batch implemented on the same day with the same batch of oviposition substrate should not be considered independent; it is pseudo-replication [81]. In order to document the baseline, including its 95% CI, it is recommended that choice experiments with different test substrates in a cage must always be implemented in parallel with a control experiment with the same number of equal choices. This validates the experimental design [37] and allows statistical comparison of the odds of success in the test experiment with the odds of success in the control experiment (baseline).

The classic oviposition index represents a proportional comparison of the numbers of eggs, egg rafts or females [82] but is rarely used in oviposition experiments with *An. gambiae* s.s.. Frequently the mean number of eggs in test and control cups is compared using classical ANOVA and *t* tests [30, 34, 35, 61, 63, 66, 67, 83–87]. These assume normality of data distribution and homogeneity of variance [88], but both assumptions are violated when looking at egg counts of *An. gambiae* s.s.. Some (log-) transform egg-counts or use non-parametric tests that do not assume a normal distribution. However, log-transforming count data for analyses have recently been challenged except when dispersion is small and means are large [89]. Moreover, non-parametric have reportedly been invalidated even by “small differences in variance and moderate degrees of skew” [90–92]. When distributions are skewed (such as for negative binomial distributions) differences in means are prone to go together with differences in variance [90]. It is also imperative to appreciate the non-independent nature of the data from control and test cups in the same cage and the dependent nature of the data derived from the same rounds when analysing choice egg-count bioassays. This violation of independent observations assumption results on downwardly biased standard error estimates, overly large test statistics, and inflated type I error rates. The statistical procedure used must, therefore, take account of that by including repeated measure terms.

It is strongly suggested analysing choice bioassays using generalized regression models that allow for the appropriate distribution to be fit to the model rather than transforming the data [88, 89]. Preference should be given to analysing proportions (of eggs laid or of females laying in test and control) using a binomial distribution than to analyse counts using a negative binomial or Poisson distribution. Importantly, these models allow including critical explanatory variables as well as random factors and/or repeated measures that might have affected the outcome. Based on the model, the effect size of the test can be reported using both odds ratios and predicted averages together with associated CIs [93].

## Conclusion

Individual *An. gambiae* s.l. lay a widely different number of eggs. A large proportion of these also skip oviposit, spreading their eggs in more than one substrate. These egg-laying patterns can lead to spurious conclusions of oviposition substrate preferences based on two choice egg-count bioassays. In order to increase the accuracy of these bioassays, designs that take into account the natural variability in the number of eggs and ensure sufficient replication are needed. Experiments are most accurate when gravid females are prepared and selected under carefully controlled conditions and when implemented in a two-tier design with 165 individual mosquitoes in each treatment arm: 165 cages each with one mosquito given a choice between a test and control solution and 165 similar cages where the mosquito has a choice between two identical control solutions. This will enable the detection of differences in substrate preferences of as little as 15% with sufficient statistical power and significance.
